# Resilience after trauma: from surviving to thriving

**DOI:** 10.3402/ejpt.v5.25339

**Published:** 2014-10-01

**Authors:** Nicole R. Nugent, Jennifer A. Sumner, Ananda B. Amstadter

**Affiliations:** 1 Department of Psychiatry and Human Behavior, Warren Alpert Medical School at Brown University and Bradley Hasbro Research Center, Rhode Island Hospital, Providence, Rhode Island; 2 Department of Epidemiology, Columbia University Mailman School of Public Health New York, NY, USA; 3 Virginia Institute for Psychiatric and Behavioral Genetics in the Departments of Psychiatry, Psychology, & Human and Molecular Genetics at Virginia Commonwealth University, Richmond, VA, USA

Resilience after trauma is one of the most compelling phenomena in contemporary traumatic stress research. To increase the understanding of resilience and its applications to policy, research, assessment, prevention, and intervention in the field of traumatic stress, professionals from across the globe gathered for the 29th annual meeting of the International Society for Traumatic Stress Studies (ISTSS) themed “Resilience after Trauma: From Surviving to Thriving.” In this thematic cluster, we highlight contributions from the plenaries from the 2013 meeting. We hope to find answers to: What is resilience? Who is resilient and what makes them so? How can resilience be fostered in the wake of trauma? What can we learn from highly resilient individuals that will help inform our work with survivors?

The first paper is based on a panel discussion chaired by Dr. Steven Southwick (Southwick et al., 2014). The esteemed panelists, Drs. George Bonanno, Catherine Panter-Brick, Ann Masten, and Rachel Yehuda, had a lively and spirited discussion about how best to approach the definition and scientific study of this important concept. Here, the panelists expand on the discussion points posed by Dr. Southwick. The second paper, written by Dr. Dennis Charney and his colleague Dr. Brian Iacoviello (Iacoviello & Charney, 2014), was inspired by Dr. Charney's moving plenary session that integrated his research on the neurobiology of resilience with real-world examples from his personal interviews with resilient individuals. The third paper is a dialog between Amanda Lindhout, a humanitarian and social activist, and Dr. Katherine Porterfield, a psychologist who has been an important part of Ms. Lindhout's treatment experiences (Lindhout & Porterfield, 2014). At the conference, Ms. Lindhout delivered a poignant personal account of her resilience in the aftermath of her kidnapping, and the related submission is inspired by questions from attendees of her talk. Amanda was a journalist in Somalia when she was kidnapped and held hostage for 460 days. After her release, she enacted the promise she'd made to herself during her captivity by founding the non-profit Global Enrichment Foundation, which provides educational and community-based empowerment programs, as well as humanitarian interventions during crisis. These plenary presentations, and the manuscripts yielded from them, will aid the field's understanding of the many potential trajectories of health following traumatic exposure, ranging from the development of persistent stress-related disorders to posttraumatic growth.

In the perceptive piece contributed by Southwick et al. (2014), Southwick and his co-authors—experts in resilience from different disciplines—tackle the challenging task of answering key questions regarding the nature of resilience. These questions do not have simple answers, and this article offers a thought-provoking discussion about the current state of the field and presents ideas about next steps to further our knowledge about fostering resilience in individuals, families, and communities affected by traumatic stressors. Each of the experts provides a commentary on their work on resilience and how it has evolved over time. These authors have employed different definitions of resilience in their own work, and many noted that the definitions they themselves use in their research have evolved over time. Nonetheless, there were a number of points of consensus among the experts. First, it was noted that a large focus of energy has been placed on understanding the underpinnings of responses to traumatic stressors that are on the negative end of the spectrum (e.g., chronic psychopathology and impairment), and by doing so, the field has neglected to also focus on the factors that give rise to resilient outcomes. Second, the complexity of this concept was uniformly agreed upon; the authors note that resilience is not monolithic, but rather is more of a continuum. They note that resilience may be domain specific, as well as culture and context specific. Third, the authors concurred that given that individuals are embedded in systems (e.g., families, religions, organizations, communities, societies), a multilevel and multidisciplinary approach must be implemented in order to fully elucidate determinates of resilience and effectively foster resilience. In addition, the authors agreed that the inclusion of novel technologies into resilience studies, such as biomarkers, has scope both for basic and intervention science.

Although not presented by the panelists, an additional perspective described in other talks and in the dialog of ISTSS attendees is that resilience and psychological symptoms are not necessarily a single continuum. During Amanda Lindhout's talk, for example, Ms. Lindhout described her daily (and sometimes moment-to-moment) decision to be present in her life and to move forward in resilience at the same time that she continues to face symptoms commonly experienced by those with posttraumatic stress disorder (PTSD). Others have argued that resilience and symptoms can coexist as independent orthogonal constructs (Luthar, Cicchetti, & Becker, 2000; Shalev & Errera, 2008; Shalev, Tuval-Mashiach, & Hadar, 2004). Indeed, one study of Jews exposed to repeated terrorism found that higher levels of posttraumatic growth were associated with probable PTSD (Hobfoll et al., 2008). Amanda Lindhout's discussion with Dr. Porterfield illustrates the idea that extreme trauma can take a real toll while the survivor may remain resilient in critically important ways.

When describing her response to serving as a journalist reporting on extreme trauma, Amanda Lindhout describes how her experiences at that time had compelled her to want to understand what “enables human survival when so much has been destroyed.” Her article provides a remarkable window into the qualities and experiences that would permit Ms. Lindhout's own survival and even resilience during, and in the years that followed, her abduction and captivity. While facing trauma, Ms. Lindhout describes using mindfulness, relaxation techniques, exercise, cognitive strategies ranging from distraction to reframing and cognitive flexibility, and social support. Ms. Lindhout also describes forgiveness as critically important to facilitating her coping and resilience both during and after her traumatic experiences. An emerging scientific literature points to the benefits of forgiveness—of others and of oneself—for survivors of a range of traumatic experiences (Hamama-Raz, Solomon, Cohen, & Laufer, 2008; Snyder & Heinze, 2005; Van Loey, van Son, van der Heijden, & Ellis, 2008; Weinberg, Gil, & Gilbar, 2013; Witvliet, Phipps, Feldman, & Beckham, 2004). Indeed, there is initial evidence supporting an intervention focused on forgiveness (Reed & Enright, 2006), and one recent intervention study framed forgiveness as a key part of “affective resolution” (Ford, Chang, Levine, & Zhang, 2013). Ms. Lindhout also provides important information for the trauma community regarding being pushed to spend 10 days detailing her experiences to a psychologist in a “debriefing.” Ms. Lindhout described having been in a vulnerable place following her release and feeling unable to refuse the interview, which she experienced as distressing both during the interview and upon later reflection. Importantly, imposed acute posttrauma “debriefing” is not supported by research or practice parameters from relevant professional organizations (Bisson et al., 2010; Forneris et al., 2013; Nash & Watson, 2012; Tol, Barbui, & van Ommeren, 2013). Of particular relevance to clinicians and researchers alike, Ms. Lindhout describes her own experiences with symptoms of PTSD as well as components of her experiences with psychotherapy. Finally, Dr. Porterfield comments on Ms. Lindhout's responses, placing them in the context of extant scientific research related to resilience and treatment for PTSD.

This cluster also includes a thoughtful contribution written by Dr. Karestan Koenen (Koenen et al., 2014) on Amanda Lindhout and Sara Corbett's book, *A House in the Sky*. In this paper, Dr. Koenen reflects on her personal reactions to *A House in the Sky*. She notes how the authors struck a delicate balance between describing the details surrounding the traumatic experiences Amanda was exposed to, while simultaneously focusing on Amanda's reactions to the events and her active and purposeful efforts to cope with the atrocities she experienced. Dr. Koenen comments on how Amanda's recovery process in the areas of both physical and mental health can inform traumatic stress professionals.

In their contribution to this special section, Drs. Iacoviello and Charney provide a nuanced and encompassing perspective on what characterizes resilience and how we can foster these adaptive characteristics in order to promote well-being after adversity. Based on an integration of findings from both empirical studies and interviews with individuals who exhibited resilience in the aftermath of severe trauma, Charney and colleagues have identified six psychosocial factors that promote resilience in individuals: 1) optimism, 2) cognitive flexibility, 3) active coping skills, 4) maintaining a supportive social network, 5) attending to one's physical well-being, and 6) embracing a personal moral compass. These factors comprise cognitive, behavioral, and existential elements, a conceptualization that has been supported by other research on the nature of resilience (e.g., Bradley et al., 2013; Connor & Davidson, 2003), and they interact with one another to encourage resilient functioning after adversity. Not only do these psychosocial factors help to identify those individuals who are most likely to exhibit resilience after trauma (e.g., Ahmad et al., 2010; Bonanno, Papa, LaLande, Westphal, & Coifman, 2004; Pietrzak & Southwick, 2011), but they also represent targets for intervention. These psychosocial factors are malleable, and Charney provides recommendations for how individuals can foster the different cognitive, behavioral, and existential components that promote resilience.

Moreover, Drs. Iacoviello and Charney describe how two empirically supported treatments for PTSD—prolonged exposure therapy and cognitive processing therapy—cultivate some of these characteristics in trauma-exposed patients (e.g., by fostering active coping). Nevertheless, existing interventions do not address all six psychosocial factors that promote resilience, and the authors highlight ways to augment current treatment protocols by specifically targeting additional factors shown to foster resilience, such as interpersonal effectiveness. Although efficacious treatments for PTSD exist (e.g., Bisson et al., 2007; Cloitre, 2009), not all individuals respond to current approaches. Expanding treatments by incorporating resilience-focused elements could be one way to increase the positive impact of psychosocial therapeutic interventions. Furthermore, specific resilience-focused training programs (e.g., Lester et al., 2011; Rose et al., 2013) may represent another promising future direction, particularly with respect to prevention. Iacoviello and Charney acutely note how resilience is not just relevant to posttrauma functioning but that it is also pertinent before individuals experience trauma. Promoting resilience with these training programs prior to trauma exposure, as well as in the immediate aftermath of trauma prior to the development of posttraumatic psychopathology, may hold potential as primary and secondary prevention measures.

In addition to describing the psychosocial characteristics that promote resilience in individuals, Iacoviello and Charney describe four qualities (i.e., social capital, community competencies, economic development, and information and communication) that characterize resilient communities (e.g., Norris et al., 2008). In doing so, they make connections between research on resilient individuals and resilient communities, and they draw parallels between the cognitive, behavioral, and existential factors that promote resilience at both the individual and the community level. In sum, resilience does not apply to just one population or context. Charney's collaborative work emphasizes how the psychosocial factors that promote resilience operate at multiple levels and are relevant both prior to and after trauma. The contributions of this work offer guidance and inspiration for both researchers and clinicians who strive to help trauma-exposed populations.

Taken together, these articles highlight the challenges in defining and measuring resilience, as well as the importance of increased scientific and clinical focus on resilience. Illustrated by the debate among the panelists, perhaps the greatest challenge ahead for the trauma community is to continue to work toward refining and even redefining our definitions of “resilience.” Researchers may wish to distinguish the dynamic process of resilience, which may change both across and within individuals over time, from a trait-like construct, although traits such as optimism or hardiness may indeed contribute to resilience during and following trauma (Luthar et al., 2000). As others have described (Luthar et al., 2000), researchers and clinicians would be well served to distinguish among domains of functioning when considering resilience; for example, a police officer might perform at a high level at work while also experiencing symptoms of PTSD that may impact other areas of life, such as social functioning. Illustrated nicely by all of the articles, the study of resilience, including understanding factors that promote resilience over the course of trauma and life after trauma, must be the central goal for researchers and clinicians in the field of traumatic stress studies.

*Nicole R. Nugent* Department of Psychiatry and Human Behavior 
Warren Alpert Medical School at Brown University and 
Bradley Hasbro Research Center 
Rhode Island Hospital 
Providence, Rhode Island *Jennifer A. Sumner* 
Department of Epidemiology 
Columbia University Mailman School of Public Health 
New York, NY 
USA *Ananda B. Amstadter* 
Virginia Institute for Psychiatric and 
Behavioral Genetics in the Departments of Psychiatry, 
Psychology, & Human and 
Molecular Genetics at Virginia Commonwealth University
Richmond, VA
USA
